# Statement der ExpertInnengruppe „Interstitielle Lungenerkrankungen und Orphan Diseases“ der Österreichischen Gesellschaft für Pneumologie zum Update der internationalen multidisziplinären Klassifikation der interstitiellen Pneumonien 2025

**DOI:** 10.1007/s00508-026-02782-0

**Published:** 2026-07-15

**Authors:** David Lang, Mathis Hochrainer, Kaveh Akbari, Luka Brcic, Klaus Hackner, Tjasa Kamenski-Rathmanner, Dagmar Krenbek, Drolaiz Liu, Patricia Marta, Anna Mayr, Felicitas Oberndorfer, Helmut Prosch, Marijan Puseljic, Georg Sterniste, Michael Studnicka, Gerlig Widmann, Mirja M. Wirtz, Markus Zwilak, Nikolaus Kneidinger

**Affiliations:** 1 Universitätsklinik für Innere Medizin mit Schwerpunkt Pneumologie, Kepler Universitätsklinikum Linz, Med Campus III., Linz, Österreich; 2 Abteilung für Innere Medizin und Pneumologie, Klinik Floridsdorf, Karl Landsteiner Institut für Lungenforschung und Pneumologische Onkologie, Wien, Österreich; 3 Abteilung für Radiologie, Salzkammergut Klinikum Vöcklabruck, Vöcklabruck, Österreich; 4 Institut für Pathologie, Landeskrankenhaus Graz II, Graz, Österreich; 5 Klinisches Institut für Pathologie, Medizinische Universität Wien, Wien, Österreich; 6 Klinische Abteilung für Pneumologie, Universitätsklinikum Krems, Karl Landsteiner Privatuniversität für Gesundheitswissenschaften, Krems, Österreich; 7 Abteilung für Innere Medizin und Pneumologie, Klinikum Klagenfurt, Klagenfurt, Österreich; 8 Institut für Pathologie und Bakteriologie, Klinik Floridsdorf, Wien, Österreich; 9 Klinisches Institut für Pathologie und Molekularpathologie, Kepler Universitätsklinikum, Johannes Kepler Universität, Linz, Österreich; 10 Universitätsklinik für Radiologie und Nuklearmedizin, Medizinische Universität Wien, Wien, Österreich; 11 Universitätsklinik für Radiologie, Abteilung für allgemeine radiologische Diagnostik, Medizinische Universität Graz, Graz, Österreich; 12 Universitätsklinik für Pneumologie, Universitätsklinikum Salzburg, Paracelsus Medizinische Privatuniversität, Salzburg, Österreich; 13 Universitätsklinik für Radiologie, Medizinische Universität Innsbruck, Innsbruck, Österreich; 14 Abteilung für Innere Medizin und Pneumologie, Landeskrankenhaus Graz II, Standort Enzenbach, Enzenbach, Österreich; 15 Universitätsklinik für Innere Medizin, Klinische Abteilung für Pneumologie, Lung Research Cluster, Medizinische Universität Graz, Graz, Österreich; 16 Klinisches Forschungsinstitut für Entzündungsmedizin, Johannes Kepler Universität, Linz, Österreich

**Keywords:** Bronchiolozentrische interstitielle Pneumonie, Gewöhnliche interstitielle Pneumonie, Alveolarmakrophagenpneumonie, Hypersensitivitätspneumonitis, Lungenfibrose, Bronchiolocentric interstitial pneumonia, Usual interstitial pneumonia, Alveolar macrophage pneumonia, Hypersensitivity pneumonitis, Pulmonary Fibrosis

## Abstract

Die Aktualisierung der American Thoracic Society/European Respiratory Society(ATS/ERS)-Klassifikation der interstitiellen Pneumonien von 2025 erweitert deren Einteilung über rein idiopathische Entitäten hinaus und umfasst nun auch Krankheitsbilder mit identifizierbaren Auslösern [1]. Zentraler Bestandteil dieser Aktualisierung ist die terminologische Neuausrichtung der Klassifikation zu einem deskriptiven, primär morphologisch orientierten Konzept mit dem Ziel der klaren Unterscheidung von radiologischen und histologischen Mustern auf der einen und von klinisch definierten Erkrankungen auf der anderen Seite. Die akute interstitielle Pneumonie (AIP) wird nun als idiopathische diffuse alveoläre Schädigung (DAD) bezeichnet, die desquamative interstitielle Pneumonie (DIP) als Alveolarmakrophagenpneumonie (AMP). Das Muster der bronchiolozentrischen interstitiellen Pneumonie (BIP) wird als eigenständiges morphologisches Muster eingeführt, der Begriff der Hypersensitivitätspneumonitis (HP) soll künftig ausschließlich der multidisziplinären Diagnose vorbehalten sein. Darüber hinaus bietet die aktualisierte Klassifikation ein biologisch begründetes und klinisch anwendbares System zur Kategorisierung in interstitielle und alveoläre Füllmuster und die auch prognostisch relevante Aufteilung in fibrotische und nichtfibrotische Verläufe. Hervorzuheben ist zudem eine stärkere Betonung der transparenten Angabe der diagnostischen Sicherheit im interdisziplinären Board für interstitielle Lungenerkrankungen (ILD-Board), inklusive der Vergabe von „provisorischen“ Diagnosen bzw. Verwendung des Begriffs der „unklassifizierbaren ILD“ bei niedriger und sehr niedriger diagnostischer Sicherheit. Die aktuelle Klassifikation kann helfen, den diagnostischen Ablauf und den Entscheidungsprozess im ILD-Board zu standardisieren und transparenter zu gestalten und dadurch auch die klinische Versorgung zu optimieren. Es ist zu erwarten, dass dieses Update die Grundlage für künftige Forschung und neue Erkenntnisse im Bereich der ILD sein wird. Daneben soll jedoch nicht unerwähnt bleiben, dass die neue Klassifikation hinsichtlich der Einführung des Musters der BIP und der provisorischen Entität der „idiopathischen BIP“ von einigen ExpertInnen kritisch beurteilt wird. Befürchtet wird konkret, dass durch die stärkere Betonung eines morphologischen Musters und die terminologische Abgrenzung zur HP die diagnostische Aufmerksamkeit für potenziell auslösende Expositionen abgeschwächt werden könnte.

**Zusatzmaterial online:**

Zusätzliche Informationen sind in der Online-Version dieses Artikels (10.1007/s00508-026-02782-0) enthalten.

## Abstract (en)

The update of the American Thoracic Society/European Respiratory Society (ATS/ERS) classification of interstitial pneumonias published in 2025 extends the oprior classification system beyond merely idiopathic entities, now also encompassing entities with identifiable triggers [1]. The core component of this update is the terminological reorientation of the classification towards a descriptive, primarily morphologically oriented concept with the aim of a clear differentiation of radiological and histological patterns from clinically defined diseases. Acute interstitial pneumonia (AIP) is now designated as idiopathic diffuse alveolar damage (DAD), and desquamative interstitial pneumonia (DIP) as alveolar macrophage pneumonia (AMP). The pattern of bronchiolocentric interstitial pneumonia (BIP) has been introduced as an independent morphological pattern, reserving the term hypersensitivity pneumonitis (HP) exclusively for multidisciplinary diagnosis in the future. Furthermore, the updated classification provides a biologically founded and clinically applicable categorization system of interstitial and alveolar filling patterns and the prognostically relevant separation into fibrotic and nonfibrotic phenotypes. Also, there is a stronger emphasis on the transparent reporting of the diagnostic confidence by interstitial lung disease (ILD) boards, including the use of “provisional” diagnoses or of the term “unclassifiable ILD” in cases of low and very low diagnostic certainty. The current classification can help to standardize the diagnostic course and to make the decision-making process in the ILD board more transparent. It is to be expected that this update will be the foundation for future research and for new knowledge in the field of ILD. In addition, it should however not be ignored that the new classification has been critisized by some experts with respect to the introduction of the pattern of BIP and the provisional entity of “idiopathic BIP”. It is feared that this change in terminology emphasizing the morphological pattern rather than causative factors could lead to reduced attention concerning potential trigger exposures in the diagnostic process.

## Einleitung

Die neue American Thoracic Society/European Respiratory Society (ATS/ERS)-Klassifikation der interstitiellen Pneumonien von 2025 basiert auf den 2002 und 2013 publizierten Versionen und entwickelt diese weiter [1–3]. Ein zentraler Beweggrund für die neue Klassifikation ist es, den Fokus über rein idiopathische Entitäten hinaus zu erweitern. Die Vorversionen sprachen noch von „idiopathic interstitial pneumonias“, doch einige früher als idiopathisch geltende Entitäten – etwa die respiratorische Bronchiolitis mit interstitieller Lungenerkrankung (RB-ILD) – lassen sich heute klaren Ursachen wie einer inhalativen Tabakrauchbelastung zuordnen. Zudem wird die zugrunde liegende Ätiologie bei vielen PatientInnen erst im Verlauf erkennbar, etwa nach erneuter Expositionsanalyse oder dem späteren Auftreten autoimmuner Merkmale. Die gemeinsame Klassifikation sowohl idiopathischer als auch sekundärer interstitieller Pneumonien bildet die klinische Realität besser ab und ermöglicht, Erkrankungen bei späterer Klärung ihrer Ursache innerhalb derselben Systematik zu belassen.

Ein weiterer wichtiger Ansatz ist die klare Trennung zwischen morphologischen Mustern in der Computertomographie und der Histologie einerseits und multidisziplinär gestellten Diagnosen andererseits. Radiologische und histopathologische Muster wie „usual interstitial pneumonia“ (UIP), „non-specific interstitial pneumonia“ (NSIP) oder das für die Hypersensitivitätspneumonitis (HP) typische Muster, nun als „bronchiolozentrische interstitielle Pneumonie“ (BIP) bezeichnet, stellen keine Diagnosen dar, sondern deskriptive Befunde, die je nach klinischem Kontext unterschiedliche Erkrankungen repräsentieren. Die Betonung dieser Unterscheidung soll verhindern, dass Muster irrtümlich direkt einer Diagnose gleichgesetzt werden. Gleichzeitig knüpft die neue Klassifikation an frühere Konzepte zum Krankheitsverhalten an und berücksichtigt, dass bestimmte Muster mit charakteristischen Verläufen und Therapieansprechen einhergehen.

Schließlich trägt die Aktualisierung der anhaltenden Herausforderung diagnostischer Unsicherheit Rechnung: Selbst nach strukturierter multidisziplinärer Fallkonferenz im ILD-Board bleiben etwa 10 % der ILD-Fälle unklassifizierbar [4]. Die neue Klassifikation fördert eine transparente Dokumentation der diagnostischen Sicherheit und akzeptiert Unsicherheit als inhärenten Bestandteil der ILD-Diagnostik. Sie fordert auch explizit dazu auf, diese diagnostische Unsicherheit im ILD-Board abzuschätzen und im Befund zu berichten.

## Interstitielle Muster

**Abb. 1:**
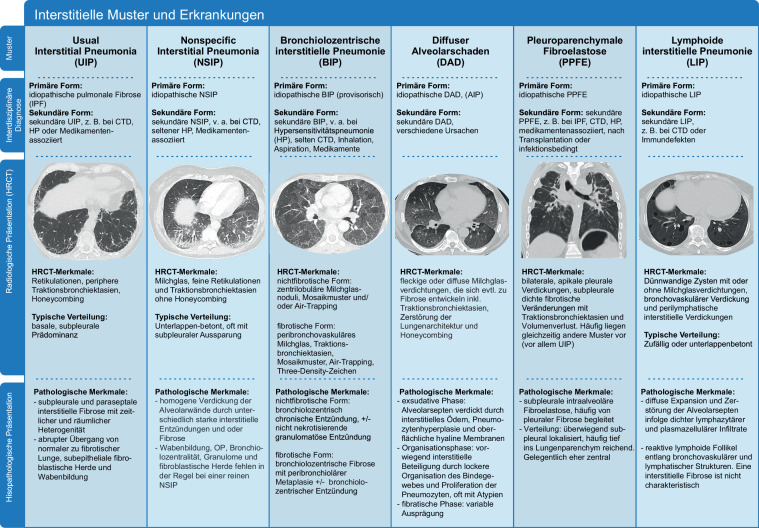
Übersicht der interstitiellen Muster

Die wichtigsten Neuerungen im Bereich der interstitiellen Muster betreffen 2 semantische Anpassungen: Erstens wird der Begriff der akuten interstitiellen Pneumonie (AIP) durch den Begriff des diffusen Alveolarschadens (DAD) ersetzt, um zu berücksichtigen, dass auch bei anderen interstitiellen Pneumonien akute Verläufe auftreten können. Zweitens definiert der neu eingeführte Begriff der BIP ein histologisches und radiologisches Muster einer prädominant bronchiolozentrischen interstitiellen Pneumonie, das sowohl entzündliche als auch fibrotische Ausprägungen umfassen kann. Dieses Muster wird v. a. bei der HP beobachtet, kann jedoch seltener auch im Zusammenhang mit Kollagenosen, Aspiration sowie inhalativ oder medikamentös bedingten Schädigungen auftreten [5]. Die Einführung des Terminus BIP soll eine klare begriffliche Abgrenzung zwischen dem morphologischen Muster und der klinischen Diagnose ermöglichen. Dadurch soll auch verhindert werden, dass ein rein radiologisches oder histologisches Muster automatisch mit einer bestimmten Erkrankung gleichgesetzt wird. Der Begriff erlaubt außerdem, sekundäre und idiopathische Ursachen voneinander zu trennen. Bei einem nicht unerheblichen Teil der PatientInnen mit BIP kann kein HP-typischer exogener Auslöser nachgewiesen werden. Die Verwendung des Begriffs idiopathische BIP als multidisziplinäre Diagnose, bei der nach einer gründlichen Untersuchung keine Ursache gefunden wurde, unterstreicht die Notwendigkeit einer aktiven, fortlaufenden Überwachung auf verschiedene mögliche Ätiologien einschließlich Kollagenosen. Die Diagnose einer HP bleibt demnach der multidisziplinären Fallbesprechung vorbehalten und sollte nicht mehr zur Bezeichnung eines rein radiologischen oder pathologischen Musters verwendet werden. Selbiges gilt auch für alle anderen ILD (Abb. 1 und 2, ergänzende Abbildung 1.

**Abb. 2:**
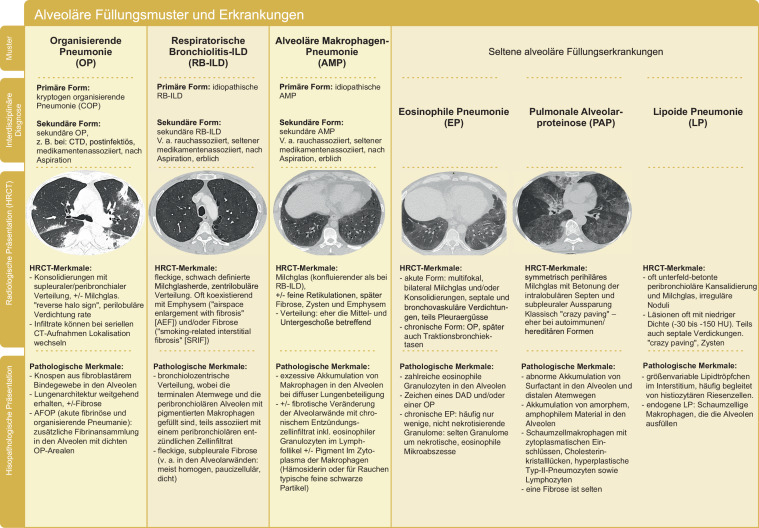
Übersicht der alveolären Füllungsmuster

## Alveoläre Füllungsmuster

Die alveolären Füllungsmuster werden in der neuen Klassifikation erstmals von den „klassisch interstitiellen“ Mustern abgegrenzt. Diese Gruppe umfasst Veränderungen, die sich in erster Linie im Lumen der Alveolen und der distalen Atemwege manifestieren und sowohl durch zelluläre als auch azelluläre Infiltration verursacht werden können. Neben diesen alveolären Hauptbefunden können begleitende interstitielle fibrotische und inflammatorische Veränderungen in variablem Ausmaß vorliegen.

Radiologisch sind flächige Milchglasinfiltrate der Leitbefund, die weitere Klassifizierung erfolgt oft in der histologischen Aufarbeitung durch Analyse des alveolären Inhalts (Abb. 2, ergänzende Abbildung 1).

Die wesentliche Neuerung der aktuellen Klassifikation ist die Einführung der „Alveolarmakrophagenpneumonie“ (AMP), welche die „desquamative interstitielle Pneumonie“ (DIP) ersetzt. Die AMP ist histologisch durch die Ansammlung von Makrophagen in den Alveolen gekennzeichnet – entgegen der früheren Annahme, dass die Erkrankung durch Abschilferung („Desquamation“) von Pneumozyten entstehen würde. Wie bei den verwandten Erkrankungen der RB-ILD und der Langerhans-Zell-Histiozytose liegt auch bei der AMP meist eine tabakrauchassoziierte Genese vor. Wichtig ist jedoch, dass idiopathische Fälle vorkommen können und sogar häufiger als bei der RB-ILD ein Nichtraucherstatus vorliegt. Weitere sekundäre Ursachen können andere inhalative Noxen sein, auch kann dieses Muster als pulmonale Manifestation von Kollagenosen (CTD) auftreten.

## Umsetzung in der klinischen Praxis

Neben den genannten Änderungen der Nomenklatur soll die neue Klassifikation die AnwenderInnen anleiten, einem standardisierten und nachvollziehbaren diagnostischen Weg in der multidisziplinären Evaluation aller PatientInnen mit ILD zu folgen.

**Abb. 3:**
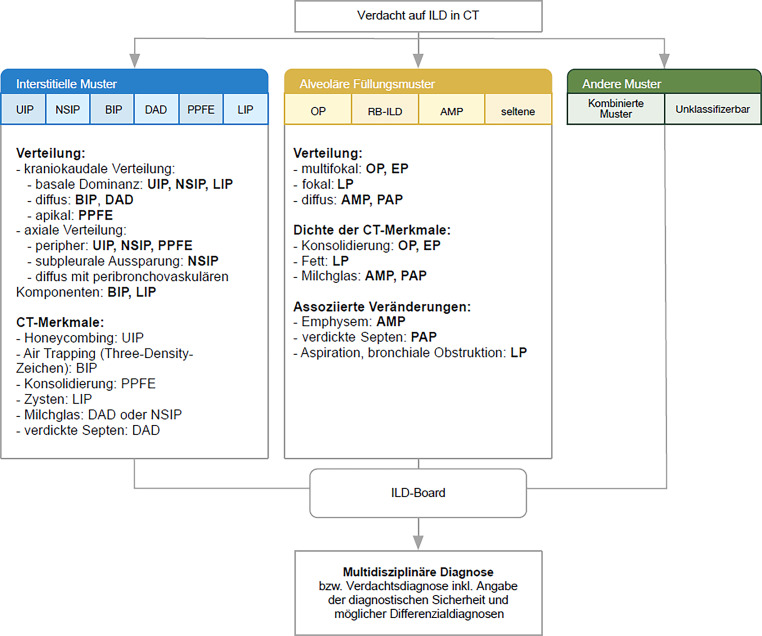
Schematischer Ablauf der radiologischen ILD-Diagnose abhängig von verschiedenen vorkommenden HRCT-Mustern

Gewöhnlich beginnt dieser Prozess mit einer CT des Thorax, in der häufig bereits ein charakteristisches radiologisches Muster identifiziert werden kann. Dieses kann der weiteren Abklärung oft schon eine entscheidende Richtung geben, beispielsweise durch eine gezielte Evaluation auf Autoimmunerkrankungen bei einem NSIP-Muster oder die differenzialdiagnostische Einordnung beim Muster einer UIP, organisierenden Pneumonie (OP) oder BIP. Nach erfolgter ätiologischer Abklärung soll dann eine Unterscheidung in eine sekundäre/ätiologisch geklärte oder in eine primäre/idiopathische Genese erfolgen (Abb. 3).

Mit der Einführung der BIP als neues morphologisches Muster, das insbesondere im Kontext der Diagnose einer HP häufig vorliegt, kann das Prinzip der klaren Trennung zwischen radiologisch-pathologischem Muster und multidisziplinärer klinischer Diagnose nun konsistent über alle Entitäten hinweg verwendet werden. Einige namhafte ExpertInnen traten jedoch in der wissenschaftlichen Diskussion über die Einführung einer möglichen vorläufigen („provisional“) Diagnose einer „idiopathischen BIP“ von ihrer Autorschaft zurück [6]. Kritisiert wurde v. a., dass die Einführung der idiopathischen BIP als provisorische klinische Entität den Stellenwert der sorgfältigen Suche nach relevanten Expositionen im Rahmen der HP-Diagnostik verringern könnte. Die AutorInnen der originalen Publikation betonen jedoch, dass die geäußerte Anpassung primär semantischer Natur sei und keinen Einfluss auf den in aktuellen Leitlinien [7, 8] empfohlenen diagnostischen Ansatz für die HP habe. Auch die AutorInnen dieses Statements sehen die Möglichkeit, dass die CT-morphologische Mustereinteilung einer BIP zur voreiligen „idiopathischen“ Diagnose mit entsprechendem Nachteil für betroffene PatientInnen führen könnte. Diesem Risiko soll primär durch verstärkte Ausbildung sowie eine Standardisierung der Expositionsdiagnostik und des diagnostischen Entscheidungsprozesses im ILD-Board entgegengewirkt werden. Auch dafür gibt die aktuelle Klassifikation mit der konsequenten Angabe der diagnostischen Sicherheit und der transparenten Darstellung von Unsicherheit mit der Möglichkeit einer „provisorischen“ Diagnose im Rahmen des ILD-Boards eine alltagstaugliche Anleitung (Abb. 3).

Eine weitere wichtige Dimension der neuen Klassifikation ist auch die Berücksichtigung des Blickwinkels der Betroffenen auf den Diagnoseprozess. In der Vergangenheit war es für PatientInnen und BehandlerInnen beispielsweise schwierig, die Argumentation für die Diagnose HP langfristig nachzuvollziehen, wenn tatsächlich kein Hinweis auf entsprechende Exposition vorlag [9]. Die Möglichkeit einer vorläufigen Diagnose der „ILD mit idiopathischem BIP-Muster“ erlaubt hier Spielraum, um einen oft jahrelangen psychischen Druck eines unerkannten Antigens auf PatientInnen zu vermeiden. Mit dem Begriff der AMP wird auch die „frühere“ DIP ein Stück weit von der Raucheranamnese getrennt und trägt somit auch dem (seltenen) Vorkommen bei NichtraucherInnen Rechnung.

Im Vergleich zu anderen pneumologischen Krankheitsbildern sind ILD selten und vielgestaltig, weshalb die Abklärung oft komplex und die Diagnose obligat multidisziplinär zu stellen ist. RadiologInnen, PathologInnen, PneumologInnen und RheumatologInnen mit einer ausgewiesenen Expertise für diese Krankheitsbilder tragen daher die hohe Verantwortung

- der korrekten Diagnosestellung nach aktuellem Stand der Wissenschaft,
- jene transparent gegenüber anderen medizinischen Fachkräften darzustellen und
- diese auch verständlich an die PatientInnen und ihre Angehörigen zu vermitteln.

Wir sehen es folglich positiv, dass die neue Klassifikation all die erwähnten Anforderungen berücksichtigt. Instrumente wie die konsequente Angabe der diagnostischen Sicherheit, die Einführung einer „provisorischen Diagnose“ und die durchgängige ätiologische Gliederung in primäre und sekundäre Formen erscheinen hier besonders geeignet. Dieses Vorgehen ermöglicht eine höhere Standardisierung, transparentes Arbeiten und Flexibilität in der Adaptation von Diagnosen an das Auftreten neuer Befunde. Wir empfehlen daher, die in Abb. 3 und 4 dargestellten Pfade österreichweit in den ILD-Boards zu integrieren: Mustereinteilung – Abklärung sekundärer Ursachen – multidisziplinäre Besprechung im ILD-Board – Angabe der Diagnose inklusive Differenzialdiagnosen, zugrunde liegendem Muster und diagnostischer Sicherheit.

**Abb. 4:**
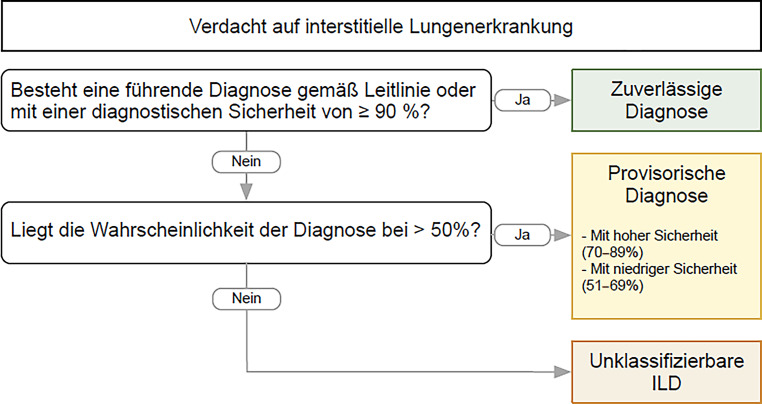
Vorgeschlagener Algorithmus zur Festlegung der diagnostischen Sicherheit im ILD-Board

## Supplementary Information

Ergänzende Abb. 1: Übersicht der histologischen Muster

## Data Availability

Alle dieser Arbeit zugrunde liegenden Daten sind in diesem Artikel enthalten.
