# Supplementary figures and images for: Statement of the Austrian Society of Pneumology expert group for interstitial lung diseases and orphan diseases on the 2025 update of the international multidisciplinary classification of interstitial pneumonias
Source: Wien Klin Wochenschr. 2026 Jul 15;138(Suppl 9):713–20. [Article in German] doi: 10.1007/s00508-026-02782-0 (PMC13395886; doi:10.1007/s00508-026-02782-0)

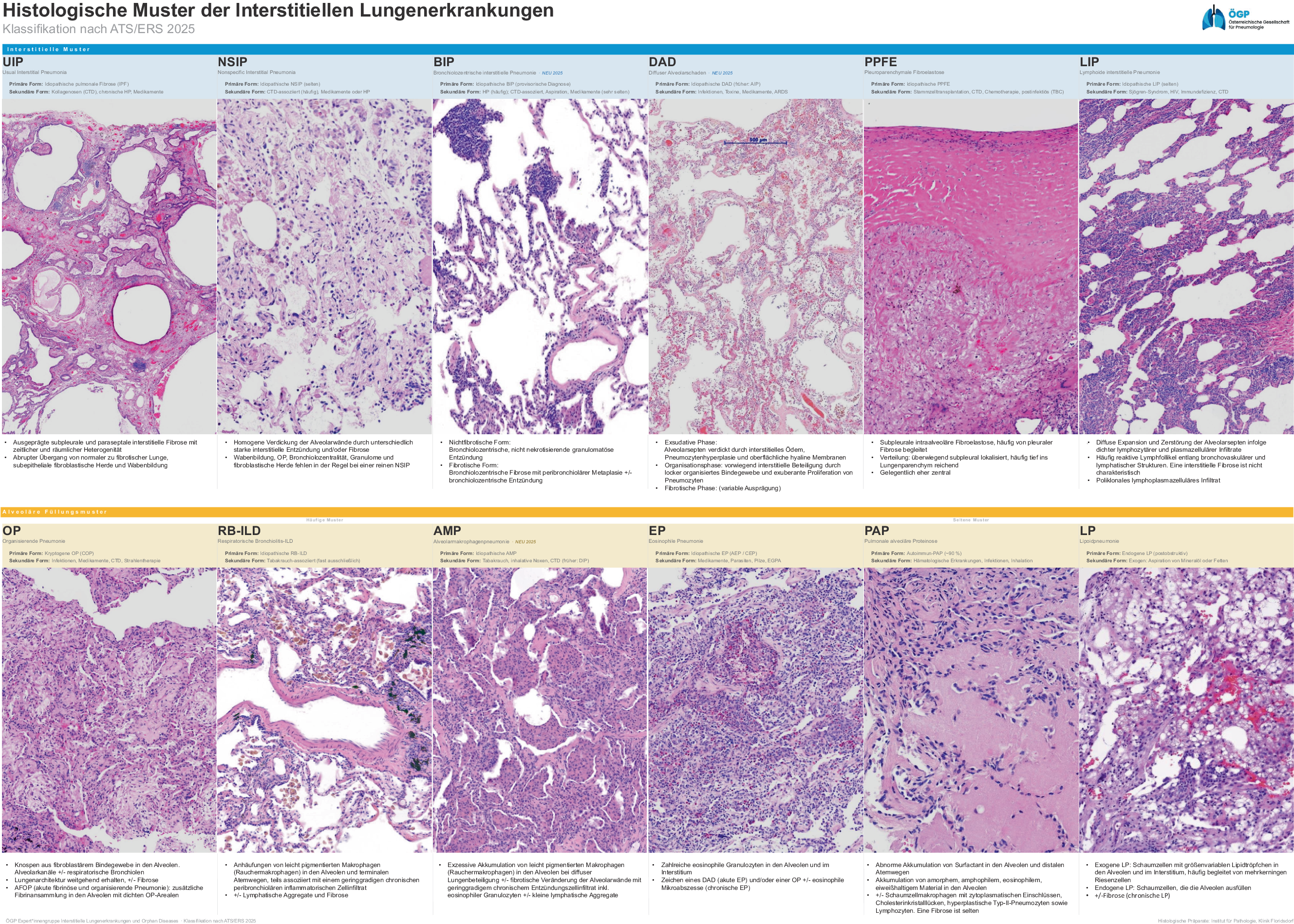

Supplement: Supplementary file 1 — Ergänzende Abb. 1: Übersicht der histologischen Muster [file 508_2026_2782_MOESM1_ESM.png]
